# More than meets the eye in Parkinson’s disease and other synucleinopathies: from proteinopathy to lipidopathy

**DOI:** 10.1007/s00401-023-02601-0

**Published:** 2023-07-08

**Authors:** Manuel Flores-Leon, Tiago Fleming Outeiro

**Affiliations:** 1 Department of Experimental Neurodegeneration, Center for Biostructural Imaging of Neurodegeneration, University Medical Center Göttingen, 37073 Göttingen, Germany; 2 Facultad de Ciencias, Universidad Nacional Autónoma de México, Ciudad Universitaria, Ciudad de México, Mexico; 3 Max Planck Institute for Multidisciplinary Science, Göttingen, Germany; 4 Faculty of Medical Sciences, Translational and Clinical Research Institute, Newcastle University, Framlington Place, Newcastle Upon Tyne, NE2 4HH UK; 5 Scientific Employee with an Honorary Contract at Deutsches Zentrum für Neurodegenerative Erkrankungen (DZNE), Göttingen, Germany

**Keywords:** Parkinson’s disease, Proteinopathy, Alpha-synuclein, Lipidopathy, Lipidostasis, Neurodegeneration

## Abstract

The accumulation of proteinaceous inclusions in the brain is a common feature among neurodegenerative diseases such as Alzheimer’s disease, Parkinson’s disease (PD), and dementia with Lewy bodies (DLB). The main neuropathological hallmark of PD and DLB are inclusions, known as Lewy bodies (LBs), enriched not only in α-synuclein (aSyn), but also in lipid species, organelles, membranes, and even nucleic acids. Furthermore, several genetic risk factors for PD are mutations in genes involved in lipid metabolism, such as *GBA1*, *VSP35*, or *PINK1*. Thus, it is not surprising that mechanisms that have been implicated in PD, such as inflammation, altered intracellular and vesicular trafficking, mitochondrial dysfunction, and alterations in the protein degradation systems, may be also directly or indirectly connected through lipid homeostasis. In this review, we highlight and discuss the recent evidence that suggests lipid biology as important drivers of PD, and which require renovated attention by neuropathologists. Particularly, we address the implication of lipids in aSyn accumulation and in the spreading of aSyn pathology, in mitochondrial dysfunction, and in ER stress. Together, this suggests we should broaden the view of PD not only as a proteinopathy but also as a lipidopathy.

## Introduction

The accumulation of proteinaceous inclusions in the brain is a common feature among neurodegenerative diseases. Proteins, often in the form of fibrillar amyloid structures, are the major components of those inclusions, and have been used to define them. Thus, neurodegenerative diseases are considered to be proteinopathies. Among these, Alzheimer’s Disease (AD), Parkinson’s Disease (PD), and dementia with Lewy bodies (DLB) are the most prevalent [34], affecting millions of people worldwide.

The histopathological hallmark of PD and DLB are inclusions enriched in α-synuclein (aSyn), known as Lewy bodies (LBs) [100, 173]. Although often ignored, LB are not only composed of proteins, but also contain a core of lipid species [7, 68] and, as recent data suggest, organelles, membranes, and even nucleic acids [166]. Interestingly, aSyn has been demonstrated to interact directly with lipids and certain membranes enriched with certain type of fatty acids [99]. Strikingly, several genetic risk factors for PD are mutations in genes involved in lipid metabolism, such as *GBA1*, *VSP35*, or *PINK1* [145]. Intriguingly, the consumption of certain fat in diets seems to have a significant impact in the development and progression of neurodegenerative diseases [79].

Although alterations in lipid metabolism and the balance of their species, known as lipid homeostasis (herein referred to as *lipidostasis*, in analogy to proteostasis), is deeply associated with neurodegeneration in PD (also reviewed in [5, 33, 39, 55, 59, 107]), the molecular mechanisms involved are still poorly understood. Nevertheless, integrated genome-wide association studies (GWAS) of PD show that several of the possible pathways implicated in PD are directly or indirectly connected with lipidostasis [103]. These include inflammation, altered intracellular and vesicular trafficking, mitochondrial dysfunction, and alterations in the protein degradation systems [41, 103]. The latter emerge from data showing that aSyn interacts with certain lipid species and that the accumulation of both occur in lysosomal storage diseases, for example Gaucher’s disease. Mutations in *GBA1*, the gene causing Gaucher’s disease, increase the risk for PD. Additionally, given that the brain is highly enriched in lipids, and these molecules can, for example, regulate neuronal membrane arrangement, function as secondary messengers, store energy and participate in neuronal signaling pathways [179], imbalances in lipidostasis might be key players in altered neuronal function and possible neurodegeneration. In this review, we discuss the recent evidence that suggests how lipid biology can play major roles in PD pathology, emphasizing the implications on the accumulation and spreading of aSyn pathology, on mitochondrial dysfunction, and on endoplasmic reticulum (ER) stress.

## Parkinson’s disease

PD is the second most common neurodegenerative disease and the first most common synucleinopathy, typically affecting people over 65 years old. Over 10 million people worldwide live with PD and this number is increasing alongside with the increase in life expectancy. Resting tremor, dystonia, rigidity, bradykinesia, and postural instability are the characteristic features of PD [100]. These features result from the progressive loss of dopaminergic neurons in the *substantia nigra pars compacta* (SN). aSyn accumulation and LB formation are major components thought to trigger several cellular pathways that lead to this neuronal loss [171, 173]. Although aging is the most significant risk factor for PD [38, 88, 142], lipid balance and their metabolism are emerging as important factors for PD, and will be discussed in the present review.

The scenario complicates considering that mutations in various genes, such as *LRRK2*, *PINK1*, *SNCA, DJ-1, VPS35*, and *GBA1*, have been implicated in familial and sporadic forms of PD [42, 44, 70, 140]. In particular, an overproduction of aSyn protein caused by duplications, or triplications of the *SNCA* locus, or point mutations in the *SNCA* gene, are associated with familial forms of PD [106, 152, 171, 199]. Even though most of these genetic alterations are either rare or confer variable risk to develop PD, they provide mechanistic insight into the molecular pathways associated with disease, especially since several have also been found associated with sporadic PD. In this sense, the overproduction of wild-type (WT) or mutant forms of aSyn has additional toxic effects, which might be independent of aggregation, for example when in contact with different lipids and through interactions with organelle membranes [36, 49, 60, 99, 126, 162].

Although tremendous progress has been made over the past decades, the precise molecular mechanisms underlying neuronal death are still unclear. Particularly, those that involve the interplay between genetic and environmental risk factors.

### Neuropathology of PD: protein and lipid deposition/alterations

The accumulation of aSyn in proteinaceus aggregates known as LBs and/or Lewy neurites (LNs) is one of the main neuropathological hallmarks of PD [100]. aSyn is a 14.5-kDa protein that is enriched in the presynaptic terminals of neurons [94, 125] and has been implicated as an important player in synaptic vesicle trafficking and dopamine release [1, 25, 50, 124, 198]. However, aSyn interacts with lipids and membranes and is present in various other tissues, including blood, where it likely performs other functions.aSyn aggregation is not limited to the SN, as aggregates can be found in other brain structures progressively many years before the symptomatology. Efforts have been made to classify the progression of the disease, based on the distribution of Lewy-pathology-in the brain [20–22].

Importantly, the morphology of LBs can vary depending on the brain structure where they occur, probably as a result of the stage the pathology, and likely representing a progressive process that is caught at a particular stage at the time of death. At the early stages of PD, aSyn staining starts as a diffuse-granular and pale cytoplasmic mark. As the pathology progresses, the staining becomes more intense and structures, referred to as Pale bodies, start to emerge. Finally, LBs appear, probably as a consequence of the peripheral condensation of the Pale bodies [190]. Given that as much as 90% of aSyn found in LB is phosphorylated at serine 129 [6, 65], it has been suggested that this form is involved in the initial stages of LB formation and PD pathology.

Initially, the LB structure was thought to consist of fibrillar aSyn [12, 22, 190] but it is becoming accepted that more components are involved in LB formation and maturation. The biochemical composition of LBs is highly complex and includes ~ 300 other proteins [115], ~ 90 of which have been confirmed by immunohistochemistry assays [190]. Furthermore, LBs were found to be enriched in lipids [68], membranous components that might come from vesicles, and fragmented organelles, as shown by several methods such as Fourier transform infrared micro-spectroscopy (FTIRM) [7], correlative light and electron microscopy (CLEM), stimulated emission depletion (STED)-based super-resolution microscopy, and laser-capture microdissection microscopy coupled to liquid chromatography-mass spectrometry (LC–MS) [166]. Particularly, this study identified that sphingomyelin and phosphatidylcholine are strongly enriched in these samples, further confirming that LBs are also composed of lipid species and membranes of organelles taken at some point from the cell. The latter study suggests that lipid species are tightly linked to LBs formation and/or maturation and might be associated to aSyn function, localization, and/or dynamics. Additionally, the fact that lipids are found in the core and are involved in LBs’ formation also suggests that lipidostasis impairment might be an important factor prior to protein deposition in PD.

Recently, metabolomics has opened a new door for potential biochemical biomarkers that may inform on the beginning of the disease, progression, or prognosis. Among these markers, lipid profiles or different species of fatty acids are emerging as potential ones based on evidence found in PD models and in patients [176, 180, 194, 201].

Lipidomic analyses of PD patient samples revealed alterations in 80 lipid species out of 200 that were analyzed in the visual cortex of PD patients in the Braak stage IV or V [32, 83, 165, 176]. The lipid species identified belong to the following major lipid families: sphingolipids (SL), glycerophospholipids and cholesterol. In the SL family, multiple species of sphingomyelin, ceramides, and gangliosides were found to be increased, while most lipids from the glycerophospholipid family were decreased. In this family, species of phosphatidylcholine, phosphatidylethanolamine, and phosphatidylinositol decreased [32], while phosphatidylserine species increased [32]. Interestingly, the primary visual cortex is affected in advanced stages of PD, and, among the non-motor symptoms of PD, visual hallucinations are one of the most common features [63, 85]. Furthermore, this alteration in lipidostasis reflects neuronal dysfunction that compromises the circuitry and may precede neuronal death. This finding was consistent with those by another group that found phosphatidylcholine, phosphatidylethanolamine, and phosphatidylinositol decreased in the SN of male PD patients [165]. Interestingly, modifying the concentration of certain lipid species, such as the synthesis of phosphatidylethanolamine, in PD models, leads to the accumulation of aSyn, to ER stress and mitochondrial dysfunction [193]. These reports further support the role of specific lipid species in PD, raising the possibility that some lipid species might be important players in the early or advanced neuropathological stages.

Studies in *postmortem* tissue and fibroblasts of PD patients revealed decreased levels of brain cholesterol, associated with a reduction in the expression of isopentenyl diphosphate isomerase and β-Hydroxy β-methylglutaryl-CoA reductase (HMG-CoA reductase), key enzymes in the biosynthesis of isoprenoids [136, 137]. Additionally, isotope-dilution mass-spectrometry analyses of the cholesterol metabolites 24S-hydroxycholesterol and 27-hydroxycholesterol in cerebrospinal fluid of PD patients revealed higher levels than in non-PD patients [16]. Strikingly, the levels of 24S-hydroxycholesterol correlate with disease duration [16]. Altogether, these and other studies suggest that lipidostasis imbalances likely play an important role in PD. While some may influence disease onset, others may act as markers of damage at later stages of the disease process.

Interestingly, altered SL metabolism and fatty acid biosynthesis have been detected in sebum of PD patients versus non-PD subjects [170]. Additionally, staining of *postmortem* brain sections from PD individuals using the lipid dye boron-dipyrromethene (BODIPY) showed that dopaminergic neurons in the SN accumulated lipids while astrocytes had a diminished lipid content [23]. This suggests lipidostasis is altered in different cell types in the brain and that this lipid alteration and accumulation seems to be specific for neurons.

### PD risk is associated with deregulation of lipidostasis

Given that almost 50% of the brain’s dry weight are lipids [24], it would not be surprising that many neurodegenerative diseases, including PD, may be heavily influenced by imbalances in lipidostasis [48], as evidenced by several genetic studies that we will discuss throughout this review. Consistently, several genes associated with increased risk of PD are involved in lipid metabolism.

GWAS in different populations identified the *GAK/DGKQ/IDUA* region as one of the top three risk loci for PD [31, 119, 138, 145, 169]. This region harbors the gene that encodes for the enzyme diacylglycerol kinase theta (DGKQ) that catalyzes the regeneration of phosphatidylinositol from diacylglycerol. This finding is consistent with reduced levels of phosphatidylinositol that are found in PD patients [32]. Transcriptomic studies found that the gene *ELOVL7*, that encodes for a fatty acid elongase, is also associated with PD [102, 116]. Furthermore, in several PD models, aSyn inclusions and toxicity are reduced upon inhibition of stearoyl-CoA desaturase (SCD) [58, 92, 186]. This enzyme catalyzes the rate-limiting step in the formation of monounsaturated fatty acids, suggesting that some lipid metabolic pathways have a tight relation with aSyn accumulation. Although no clear mechanism on how these genes might be involved in PD pathogenesis have been uncovered, it is important to highlight that additional genetic risk factors that involve lipid metabolism are being identified.

Fatty acids are not only important as membrane components or energy sources, but also serve as donors for post-translational modifications (PTMs). A mechanism that is dependent on specific lipid species, in this case palmitic acid, is protein palmitoylation. Palmitoylation can regulate the localization and interaction between proteins with lipid membranes, and between proteins in the same lipid domains and organelles [75, 120]. In a recent study, the palmitome of PD patients was characterized, and identified an increase in the palmitoylation of several proteins that interact with PD-associated proteins (LRRK2, DJ-1, GBA1 and aSyn) when compared to control subjects. Additionally, these proteins were found to be part of pathways associated to inflammation, cytoskeletal architecture, and mitochondrial dysfunction [30]. This suggests that lipid overload, particularly palmitic acid, may lead to excessive protein palmitoylation that might affect interaction among proteins involved in neuronal dysfunction contributing to PD onset and progression.

Perhaps the strongest direct genetic connection is that linking *GBA1* mutations with sporadic forms of PD. Glucocerebrosidase (GCase), the enzyme encoded by the *GBA1* gene, regulates SL metabolism, further supporting the view that certain lipid species likely play a role in PD onset and progression. This is the topic of the next section.

## *GBA1* mutations and SL metabolism alterations as a risk factor for PD

**Fig. 1:**
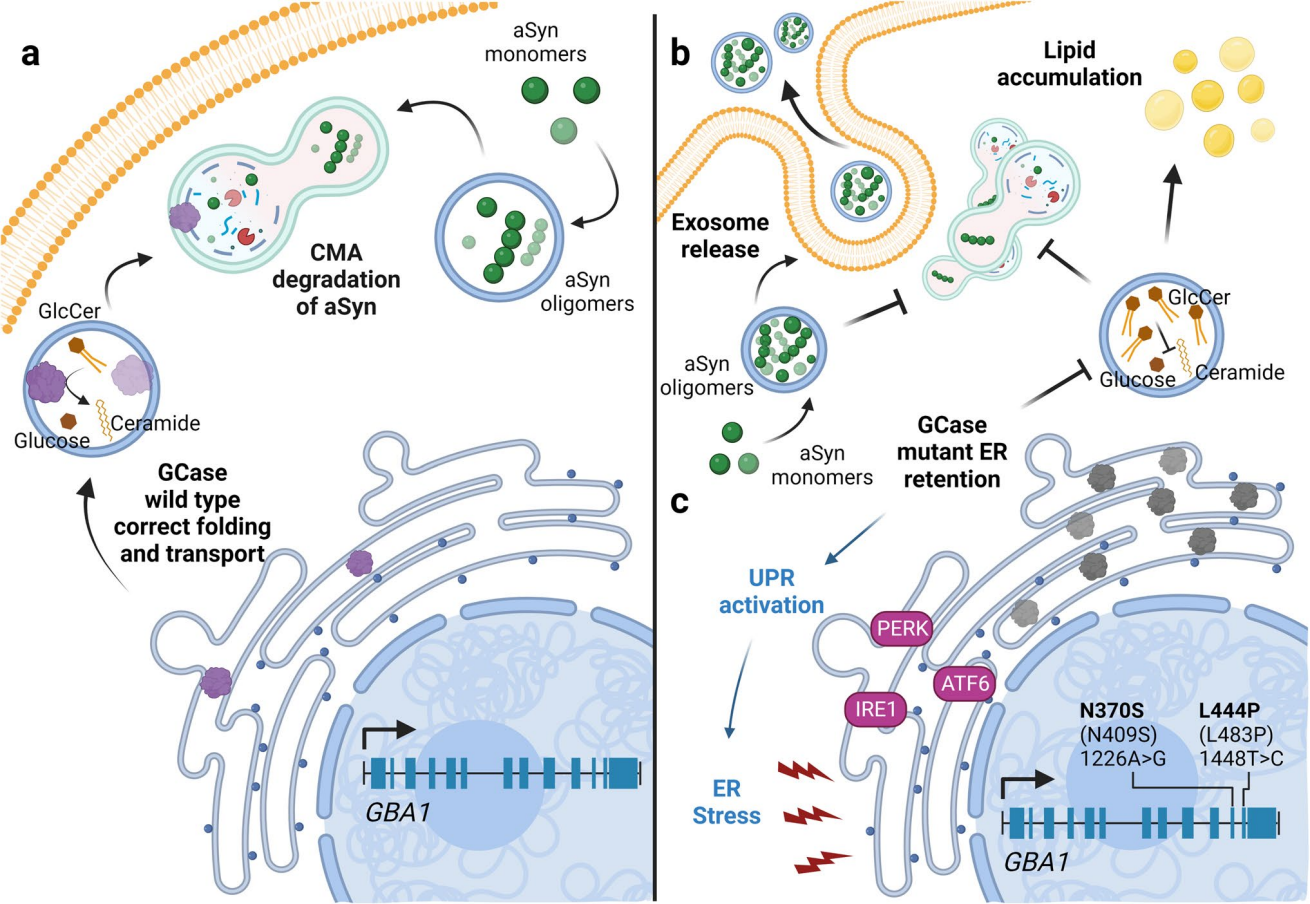
Putative loss- and gain-of-function effects of GCase mutations. **a** *GBA1* encodes for GCase. Wild type enzyme (purple protein) is correctly folded and can be transported to the lysosomes (blue complete circles) where it hydrolyzes glucosylceramide (GlcCer) into glucose and ceramide. This contributes to the correct function of the autophagic system which, through the Chaperone Mediated Autophagy (CMA) pathway, is able to degrade proteins and prevent their accumulation, for example aSyn. **b** In the loss-of-function hypothesis due to *GBA1* mutations, unfolded GCase cannot be transported to the lysosome, sphingolipid metabolism is compromised and GlcCer is accumulated. This also impairs the formation of autophagolysosomes, promoting the accumulation of aSyn oligomeric forms inside the cell. To reduce aSyn burden, changes in exosomal-mediated release of aSyn may take place. **c** In the gain-of-function hypothesis, the retention of mutant GCase in the ER activates the UPR response proteins (PERK, IRE1 and ATF6), generating ER stress which may, in turn, alter lipidostasis

*GBA1* mutations are the most common genetic risk factor for PD, increasing the risk by approximately fivefold [35, 148, 168]. GCase resides in lysosomes and is an important regulator of SL metabolism. The catabolic reaction of GCase results in the hydrolysis of glucosylceramide into glucose and ceramide [72, 164] (Fig. 1a). Homozygous loss-of-function mutations lead to a lysosomal storage disease called Gaucher’s Disease (OMIM 606423). Gaucher’s Disease patients can be classified into five types (1, 2, 3, perinatal lethal, and cardiovascular) according to substrate accumulation and neuronal affections. Type 2 and 3 patients show a degree of neurodegeneration and neuropathic manifestations that resemble clinical features of PD (reviewed in [66]). Initially, this suggested that GCase deficiency degree could be an important mechanism involved in PD.

Consistently, *GBA1* heterozygote mutations (haploinsufficiency) are associated with increased PD risk. N370S, associated with mild risk, and L444P, associated with higher risk, are the most common ones [8, 52, 69, 130, 168]. Patients carrying *GBA1* haploinsufficiency mirror sporadic PD patients to a large extent (reviewed in [161]). Nevertheless, the onset is approximately 5 years earlier, and there is a faster progression of motor and cognitive impairment when compared to sporadic PD patients [35, 52, 69, 168]. Additionally, the levels and activity of GCase are decreased in PD brains [71, 91, 132, 135, 155], leading to altered SL metabolism. Strikingly, there is a decrease in GCase activity in normal aging that reaches the levels found in PD patients, alongside with the accumulation of glucosylsphingosine in the SN [110, 155]. This suggests that alterations of the SL metabolism might be an important component of PD neuropathology, not only in carriers of *GBA1* mutations but also for sporadic PD patients where age-associated reduction in GCase activity might contribute to the onset of the pathology.

The precise mechanisms by which mutant GCase mutations increase PD risk are still unclear. There is evidence supporting both loss- or gain-of-function hypotheses (reviewed in [98]). The loss-of-function is due to defects in the correct folding of the enzyme, which leads to disrupted transport of GCase to the lysosome and a concomitant accumulation in the ER [139, 156, 164] (Fig. 1b). This alteration in GCase localization leads to the accumulation of SLs, such as glucosylceramide and glucosylsphingosine [9, 77, 91]. Interestingly, some lipid species (sphingomyelin, ceramide and monohexosylceramides) have been found increased in the plasma of PD patients [77] and, importantly, their physiological role is not only structural but also of high importance for cellular processes like autophagy, senescence, and inflammation, among others [2, 19, 93]. In the proposed loss-of-function mechanism, the reduction in GCase enzymatic activity also affects the protein degradation systems through impairment in lysosomal function and recycling [123, 153], which leads to impaired aSyn clearance and, consequently, to its accumulation [139]. Moreover, the accumulation of glucosylceramide affects aSyn aggregation by stabilizing soluble aSyn oligomers and also by inducing aggregation [127, 149] (Fig. 1b). This creates a pathogenic loop that further disrupts GCase stability and folding, fueling additional aSyn accumulation. Interestingly, when GCase mutants are overexpressed or wild type GCase is inhibited by pharmacological strategies there is an increase in the release of exosomes that contain aSyn [29, 98, 146]. In contrast, overexpression of wild type GCase results in a decrease in exosome secretion [146]. This suggests that the reduced activity of GCase contributes to aSyn spreading pathology [11, 98, 110, 131] (Fig. 1b).

Although the loss-of-function hypothesis is valid and plausible, recent results from clinical trials suggest that therapeutic approaches that overexpress wild type GCase or try to correct its folding may not be completely suitable for PD patients, particularly since *GBA1* mutations in PD patients are heterozygous (reviewed in [17]). An alternative is the gain-of-function hypothesis, whereby the retention of misfolded GCase in the ER would be responsible for lysosomal dysfunction, but through ER stress, and activation of the unfolded protein response (UPR) [61, 109] (Fig. 1c). Interestingly, the degree of GCase retention in the ER is influenced by the mutation, and this has been correlated with the severity of the pathology in Gaucher’s disease [156], and this is consistent with the reports that show that different mutations cause different risk degrees for PD.

Importantly, although there is evidence supporting loss- and gain-of-function hypotheses, one may be the result of the other. This is most likely the case in PD patients carrying *GBA1* mutations [146] (Fig. 1b, c).

Further evidence linking SLs to PD involve other enzymes that participate in this particular type of lipid metabolism. Ceramides and sphingomyelin are increased in the brain of PD patients [32, 165]. The accumulation of these metabolites correlates with an increase in the expression of genes that encode enzymes involved in the biosynthetic pathway, such as Serine palmitoyl transferase long chain base subunit 2 (*SPTLC2*), degenerative spermatocyte homolog 1 lipid desaturase (*DEGS*), sphingomyelin synthase 1 (*SGMS1*), and UDP-galactosyltransferase 8A (*UGT8A*) [32]. Another study performed in the plasma and CSF of PD patients showed that several lipid species are altered, particularly those involved in the SL metabolism [176]. Nevertheless, since SL biology is highly complex, it will be important to explore further ramifications of the pathway in order to understand how they relate to PD [111].

## The role of lipidostasis in aSyn pathology

Although progress has been made in identifying neuropathological markers of PD, the molecular and cellular mechanisms that lead to them are still unclear. Strikingly, lipid biology alterations seem to be an important player in most of the described mechanisms, particularly due to their pleiotropic functions in cellular physiology. Thus, it is important to understand how alterations in lipid species may directly affect key proteins in PD, such as aSyn, and also how such alterations impact organelles, such as mitochondria and ER, which are lipid-rich compartments that have been identified as important players in PD onset and progression.

In cells, aSyn is thought to exist primarily as a monomer [178] and, in some situations, as aggregation-resistant tetramers [13]. In pathological conditions it can be found as oligomers or fibrils. Structurally, aSyn is composed of three regions as folows: an N-terminal region that can fold into an amphipathic α-helical structure, and that binds to lipid membranes and vesicles; a central hydrophobic domain that can fold into β-sheets, the main domain responsible for its aggregation propensity; and an acidic and highly disordered C-terminal domain. Particularly, the N-terminal domain preferentially associates with glycosphingolipids (usually containing sulfate, phosphate, or sialic acid) in the membrane of synaptic vesicles [97]. This interaction is important to promote the formation of the SNARE complex between two membranes and the concomitant vesicle docking [121]. Additionally, this domain can interact with apolipoproteins, such as apolipoprotein E (ApoE), which have been implicated in increased risk for PD and DLB when the *APOE4* allele is present [18, 56, 181]. This is mediated by an increase in the aggregation propensity of aSyn when *APOE4* is present compared to other *APOE* isoforms [54]. It is interesting to highlight that most point mutations in the *SNCA* gene fall at the N-terminal domain of aSyn, the lipid interacting region, affecting the protein’s secondary structure and its lipid binding properties [28, 97, 150]. Evidence shows that aSyn interacts with lipids through several domains and that point mutations in these regions, or risk alleles involved in lipid metabolism, affect aSyn aggregation propensity.

The hydrophobic domain can also regulate the affinity of aSyn for lipid membranes [53, 67]. In this sense, it has been demonstrated a six-fold increase in the interaction between aSyn and the inner plasma membrane when gangliosides are enriched in this membrane [124]. Strikingly, a ~ 20% reduction in the levels of gangliosides is observed in PD patients [165]. This suggests that aSyn might lose some of its plasma membrane affinity, detaching and gaining aggregation properties. Additionally, it has been proposed that lipid arrangements in the membranes can induce conformational changes in aSyn amphipathic α-helical structure [62], further supporting the idea that aSyn structure and aggregation propensity could be modulated through membrane lipid composition. The evidence on how membrane lipid composition affects aSyn affinity highlights its relevance as a regulator of aSyn conformation.

Even though aSyn is traditionally seen as a presynaptic protein involved in vesicle trafficking, other functions, and interactions with membranes of other organelles, are emerging [64, 175].

### Interplay between lipid droplets and aSyn aggregation

Sterol esters and triglycerides (neutral lipids) [96, 167, 200] can be stored in the core of highly dynamic organelles called lipid droplets (LD). These organelles are composed of a phospholipid monolayer, coating proteins (such as perilipins), and enzymes [143]. A protective role against lipotoxicity is attributed to LD, due to their storing capacity during periods of nutrient surplus where harmful lipid species might be consumed/synthesized [95, 141, 163].

In a diverse range of PD models, from yeast to human cell lines, overexpression of aSyn is accompanied by an accumulation of LD [74, 144, 172, 192]. Studies in primary cortical neurons demonstrated a tight connection between aSyn toxicity, lipids, and LD, where high concentrations of oleic acid were associated with increased aSyn inclusion formation. Furthermore, if LD biogenesis is prevented aSyn toxicity increases [58]. Interestingly, in cells exposed to fatty acids, aSyn translocates from the cytoplasm to the membrane of the LD [37]. Even overexpression of selected aSyn mutants, like the A53T, induce an increase in LD accumulation [160]. This suggests that there is a connection between the excess of free lipid species in the cytoplasm and aSyn inclusion formation and toxicity. This relationship is likely bidirectional, as lipids seem to be key contributors for aSyn toxicity and, in turn, physiological levels of aSyn maintain lipid homeostasis.

### The role of lipidostasis in the life cycle of aSyn

The degradation and recycling of monomeric aSyn is thought to occur via chaperone-mediated autophagy, in the lysosome, and via the proteasome/ubiquitin system [109, 127, 128]. Once aggregates are formed, aSyn degradation takes place via macroautophagy. Several mechanisms are triggered to avoid further accumulation and toxicity, like the induction of heat shock proteins, such as HSP70, in order to stabilize soluble forms of aSyn [46, 104, 122].

Several studies suggest that GCase mutants lead to aSyn accumulation in lysosomes [43] and, as a consequence, to increased cellular release [45, 61, 151, 197], to avoid further aggregation. This mechanism contributes to the hypothesis of the prion-like spreading of aSyn pathology. This aggregation and spreading of aSyn is exacerbated in the presence of certain gangliosides, GM1 and GM3, which are also found in exosomes. This might be related to the reduced levels of gangliosides found in PD patients. Interestingly, phospholipase D1 can activate the autophagic flux, preventing the accumulation of aSyn and this enzyme is downregulated in patients with DLB [10]. This suggests that lipidostasis plays an important role in aSyn accumulation and release [4, 45, 177], saturating other neurons and disrupting their cellular machinery and function [61, 87] and contributing to the spreading of aSyn pathology.

Interestingly, aSyn accumulation has not only been reported in synucleopathies or in Gaucher’s disease. Mutations in genes that encode enzymes that are part of lipid metabolism in the lysosomes lead to diseases such as Fabry’s disease, Krabbe’s disease, and Niemann-Pick disease type C1. In these disorders, in addition to aSyn accumulation, there is also accumulation of certain SL species. Furthermore, these lysosomal storage diseases increase the risk for developing PD (reviewed in [80]). Again, this suggests that alterations in lipidostasis are associated with the accumulation of lipid-binding proteins, such as aSyn, and that such lipidic alterations might be important neuropathological alterations prior to the onset of proteinopathy.

## Lipidostasis alterations as a key player in mitochondrial impairment and ER stress

**Fig. 2:**
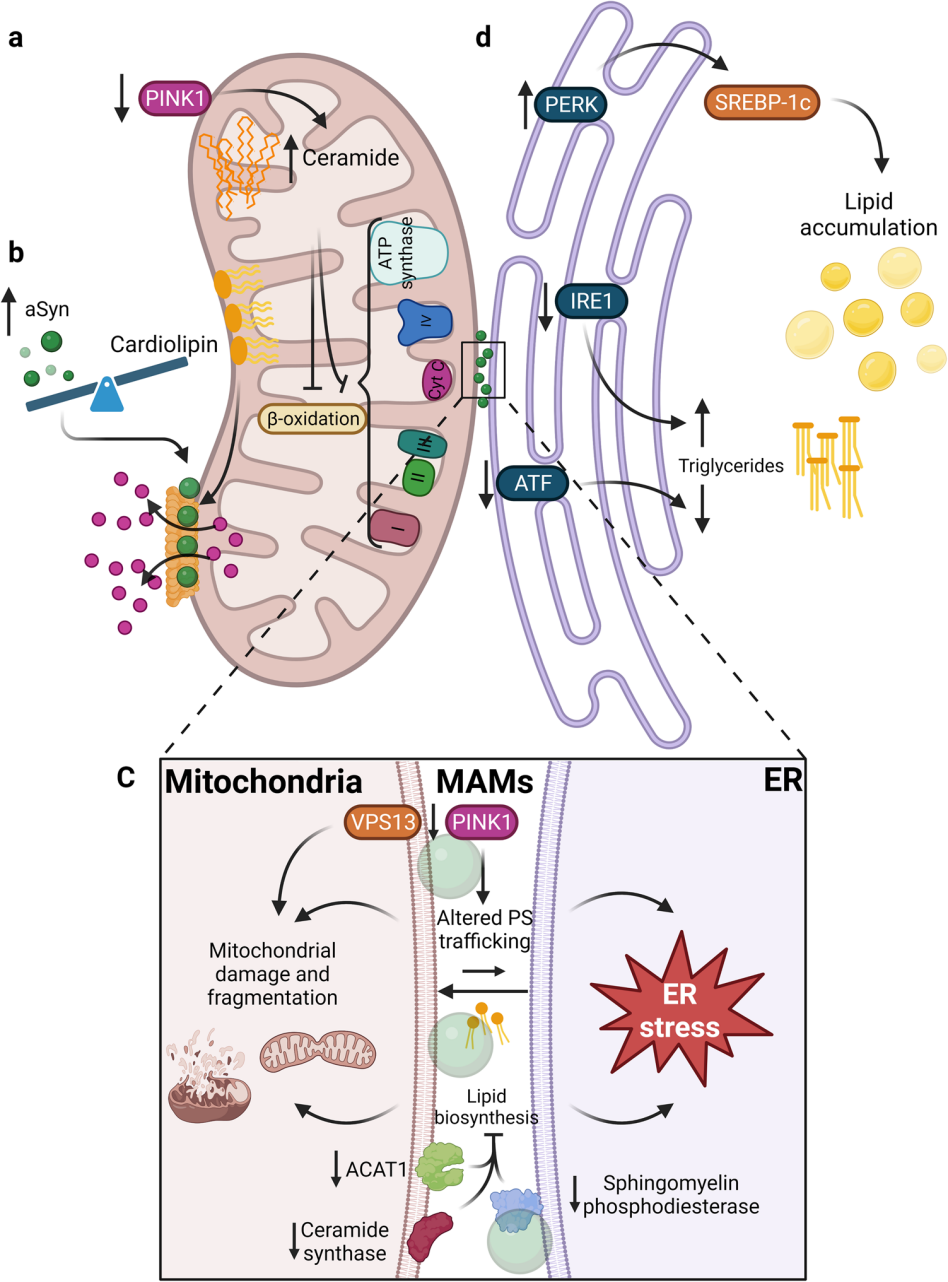
Mitochondrial dysfunction, ER stress and alterations in membrane contact sites (MAMs) are related to lipidostasis alterations. **a** Depletion or mutations in PINK1 are associated with increased ceramide levels, thereby altering beta-oxidation and the electron transport chain. **b** When the balance between aSyn and cardiolipin is altered, favoring the accumulation of aSyn, aSyn associates with the cardiolipin on the mitochondrial outer membrane inducing the formation of pores that release cytochrome c (Cyt c). **c** When proteins related to lipid metabolism, such as ACAT1, sphingomyelin phosphodiesterase, ceramide synthase or VPS13 are downregulated, processes such as lipid biosynthesis and phosphoserine trafficking are affected. Additionally, the reduction in the levels or mutations in PINK1 are also associated with altered phosphoserine trafficking between organelles. All of them lead to altered lipidostasis in the MAMs, contributing to mitochondrial dysfunction and ER stress. **d** When PERK is overexpressed, SREBP-1c is activated leading to lipid accumulation. A reduction in IRE1 and ATF leads to increased or diminished triglyceride content, respectively. Therefore, the UPR pathways in the ER can modulate and contribute to alterations in lipidostasis

Several PD genes, such as *PINK1* and *VPS13*, establish a direct bridge between lipidostasis and mitochondria [47, 101, 114, 138, 185]. PINK1 is a mitochondrial serine/threonine kinase that, when accumulated in the outer membrane of the mitochondria, phosphorylates Parkin to induce mitophagy [105]. Several PD *PINK1* deficient models display ceramide accumulation in mitochondria, negatively affecting the electron transport chain and reducing the β-oxidation rate (Fig. 2a) [133, 188]. These effects can be ameliorated when ceramide levels are lowered, or by induction of β-oxidation [188]. The lack of PINK1 is also associated with increased mitochondrial-ER contacts that cause abnormal lipid trafficking, leading to a depletion in phosphatidylserine from the ER (Fig. 2c) [189]. Furthermore, if fatty acid synthase is inhibited in PINK1 deficient models, the toxicity caused by excess in fatty acid synthesis is reduced considerably. Additionally, the inhibition of the fatty acid synthase also lowers palmitate levels and increases cardiolipin, rescuing the defects in complex I of the electron transport chain [184]. A study using a cohort of Spanish patients harboring heterozygous mutations of PINK1 revealed the presence of LBs in the brainstem and SN, and neuronal loss in the SN [159]. These features mirror those found in sporadic PD patients, suggesting that similar mechanisms might be behind the neuropathological features of PD and, again, highlighting the idea that alterations in PD-associated proteins may lead to a disruption in lipidostasis.

Mitochondrial membranes have a high content of cardiolipin [129] and, due to this glycerophospholipid, the binding affinity of aSyn to neuronal mitochondria is enhanced [27, 147, 182]. One of the first effects observed due to this enhancement is the formation of ion-permeable pores that allow the release of cytochrome c (Fig. 2b) [73]. Nevertheless, cardiolipin is also important and beneficial for aSyn refolding, preventing aggregation in some studies [158]. This suggests aSyn might be involved in the loss of mitochondrial integrity in a mechanism that is dependent on the balance between aSyn and cardiolipin.

Mitochondria and the ER communicate through physical contacts known as mitochondria-associated membranes (MAMs), which are enriched with lipids and proteins that regulate processes such as lipid synthesis and trafficking, autophagy, the unfolded protein response (UPR), redox states, among others [157]. aSyn can associate with the MAMs since it preferentially binds to membrane domains with a high composition of acidic phospholipids. However, mutations in aSyn (A30P and A53T) decrease the association with to the MAMs, thereby impairing organelle function [76].

The *VPS13* locus encodes 4 proteins (VPS13A, VPS13B, VPS13C, VPS13D) involved in the phospholipid exchange through the aqueous environment from one bilayer to another [183]. These lipid transfer proteins localize to different contact sites between organelles [26]. VPS13A, VPS13C, and VPS13D are localized at the MAMs (Fig. 2c), at the ER, and in the endolysosomal system [78, 108]. When their expression is altered lipid composition changes [82], contributing to altered organelle function (ER stress and mitochondrial dysfunction).

MAMs also play an important role in lipid homeostasis and LD biogenesis. The enzyme acyl-CoA cholesterol acyltransferase (ACAT1), which is in charge of the conversion of free cholesterol into cholesteryl esters, is enriched and has higher enzymatic activity in the MAMs than in the ER [154]. The same has been observed for enzymes important for ceramide biosynthesis, such as ceramide synthase and sphingomyelin phosphodiesterase [15, 195]. Inhibition of these enzymes leads to a relocation of the characteristic proteins of the MAMs [84], suggesting that lipid metabolism is important in maintaining these contact sites (Fig. 2c). Thus, alterations in lipidostasis causing dysfunction of the MAMs are associated with mitochondrial fragmentation [76], ER stress, and presumably even with LD biogenesis and maintenance [157]—dysfunction of all of these organelles have been observed in PD.

The ER plays a crucial role in lipid metabolism since this is the compartment where most of the lipids are synthetized, particularly membrane lipids and neutral lipids [57, 129]. Another role of the ER is to prevent the accumulation of lipids to avoid lipotoxicity [81, 174]. Additionally, the ER contains chaperones and proteins that respond to fluctuations in proteostasis, inducing a response known as the UPR in conditions of stress [40, 51]. This clearly suggests a close association between lipidostasis and proteostasis in the ER, and that impairments in either or both networks may be related to a variety of cellular problems, including those linked to neurodegeneration [89, 187]. First, a connection between aSyn and the UPR was established in a neuronal model derived from induced pluripotent stem cells obtained from a patient with a *SNCA* triplication. Neurons containing an increased aSyn load displayed an activation of IRE1/XBP1 compared to the isogenic cell line. Additionally, the presence of pIRE1α, pPERK, and pIF2a was found in neurons of PD patients that also contained LBs [86, 90], further confirming the activation of the UPR when neurons express increased levels of aSyn. Second, it is hypothesized that lipid perturbations may trigger ER stress and activate the UPR response through three known pathways: ATF6, IRE1, and PERK (Fig. 2d) [81]. Evidence supporting that alterations in lipidostasis are tightly linked with the UPR response has been obtained in non-neuronal tissues. For example, it was demonstrated that when *Ire1α* is deleted, an excess of triglycerides is detected in hepatocytes [191]. Furthermore, XBP, an important component of the IRE1 pathway, has also been demonstrated to be involved in lipogenesis [113]. Compared to the IRE1/XBP pathway, overexpression of PERK has been associated with overactivation of SREBP-1c, leading to lipid accumulation [112]. Interestingly, transgenic *Atf4-/-* mice show a minor accumulation of triglycerides compared to wild type mice when fed with either a high-carbohydrate or high-fructose diet [117, 196]. However, besides aSyn accumulation, alterations in lipidostasis may also trigger ER stress and further contribute to protein aggregation in neurons.

In total, and although additional studies will be necessary to firmly establish the role of lipidostasis in neurodegeneration, the findings above clearly demonstrate that several components of the lipid metabolic network are tightly linked to known PD-related proteins, suggesting that modulation of lipid species may constitute valid strategies for therapeutic intervention.

## Concluding remarks

**Fig. 3:**
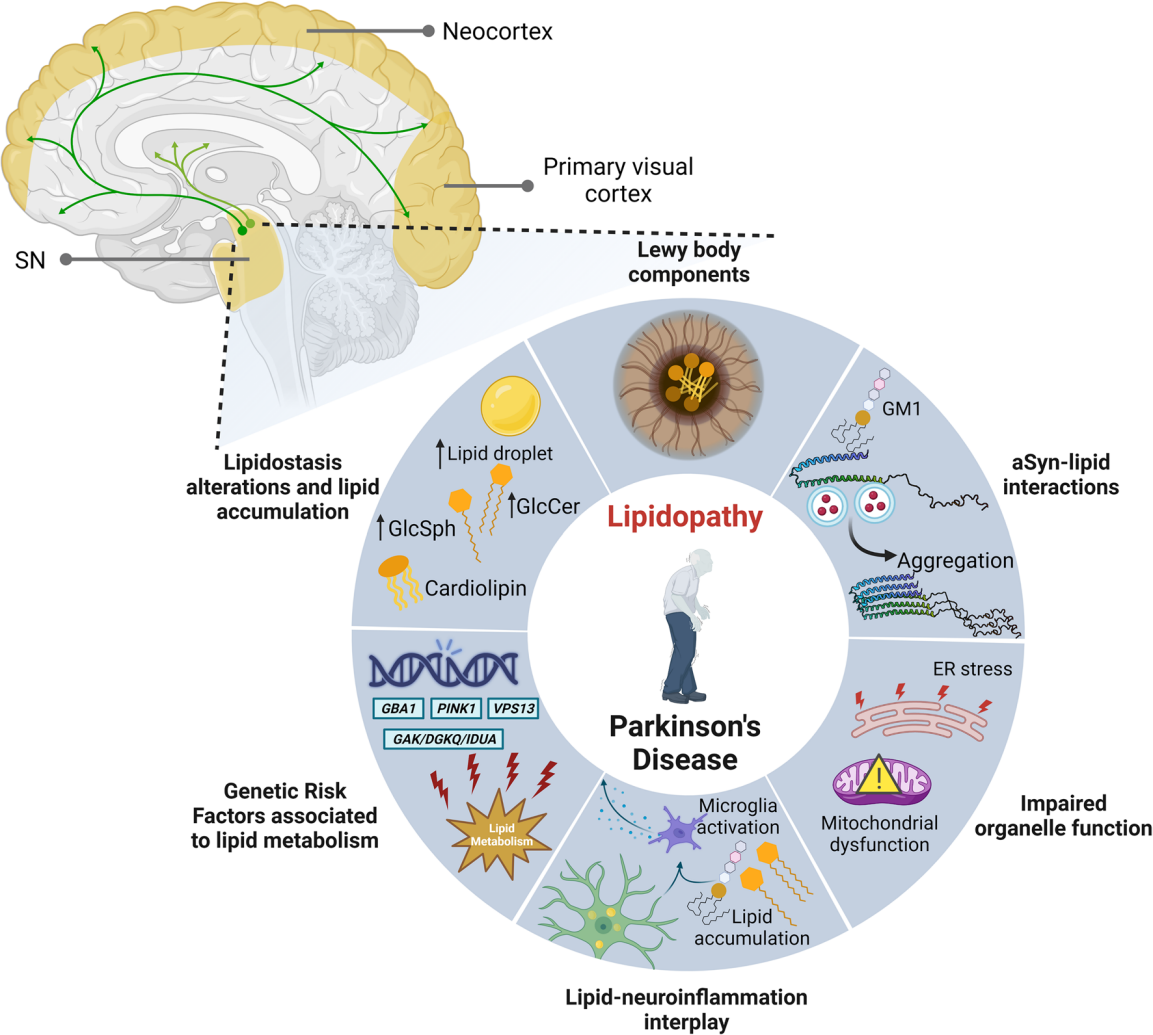
PD and synucleinopathies as lipidopathies. Alterations in lipidostasis have been observed in several brain regions (highlighted in yellow) that are also affected by the spreading of aSyn pathology (green pathways with arrows). Given that: (i) lipids/membranes are core components of LB; (ii) that aSyn structure and lipid-binding properties are affected by the proportion of lipids in organelles; (iii) that lipidostasis alterations are linked to impaired organelle function; (iv) that neuronal lipid accumulation and high concentration of lipids in the parenchyma are associated with microglial activation and neuroinflammation; (v) that several genes involved in lipid metabolism have been identified as genetic risk factors for PD and progression; and (vi) that there is a general alteration in lipidostasis leading to accumulation of particular lipid species, we posit that these diseases should be considered not only proteinopathies but also lipidopathies

PD and related synucleinopathies have been traditionally classified as a proteinopathies due to an imbalance between protein synthesis and degradation systems that lead to misfolding and accumulation of aSyn, and to a concomitant neuronal dysfunction and death. However, a fresher view into genetic, epidemiological, and mechanistic data, has brought lipidostasis into the spotlight. This idea is also fueled by the limited success in clinical trials focusing on the traditional view of synucleinopathies purely as proteinopathies, which calls for critical reconsideration of the hypotheses being tested, in the hope that greater progress can be made in the coming years. In this context, lipidostasis alterations are an emerging and exciting area. Strong evidence suggests that membrane lipids are of high importance for aSyn biology/pathobiology, contributing to aSyn fibrilization and accumulation in laboratory models. Strikingly, aSyn-lipid interactions are likely an important component in LB formation and, possible also for spreading of pathology. In summary, lipids are emerging as major contributors and drivers of PD (Fig. 3) given the following:

- Several genes involved in lipid metabolism have been identified as genetic risk factors for PD onset and progression.
- Lipids are abundant components of LB.
- aSyn structure and lipid binding is affected by the membrane composition.
- Lipidostasis imbalances are linked to impaired organelle function, such as mitochondrial dysfunction and ER stress.
- Alterations between aSyn-lipid interactions impact on organelle function.
- aSyn accumulation alters lipid droplet homeostasis.
- SLs and long-chain ceramides have been implicated in pro-inflammatory processes (reviewed in [3, 14, 19, 80, 118, 134]), consistent with the growing role of neuroinflammation and immune response in PD.

In conclusion, since lipid imbalances are emerging as an important driver of neurodegeneration, we posit that a better understanding of how alterations of lipidostasis contribute to neuropathology in PD and in other synucleinopathies will open novel avenues for therapeutic intervention and, perhaps, also for the development of novel disease biomarkers.
